# MicroRNA profiles involved in trifluridine resistance

**DOI:** 10.18632/oncotarget.18078

**Published:** 2017-05-23

**Authors:** Kenta Tsunekuni, Masamitsu Konno, Ayumu Asai, Jun Koseki, Takashi Kobunai, Teiji Takechi, Yuichiro Doki, Masaki Mori, Hideshi Ishii

**Affiliations:** ^1^ Department of Gastroenterological Surgery, Osaka University Graduate School of Medicine, Osaka, 565-0871, Japan; ^2^ Department of Medical Data Science, Osaka University Graduate School of Medicine, Osaka, 565-0871, Japan; ^3^ Translational Research Laboratory, Taiho Pharmaceutical Co, Ltd, Tokushima City, Tokushima, 771-0194, Japan; ^4^ Department of Frontier Science for Cancer and Chemotherapy, Osaka University Graduate School of Medicine, Osaka, 565-0871, Japan

**Keywords:** colorectal cancer, trifluridine, 5-fluorouracil, let-7d-5p, drug resistance

## Abstract

Trifluridine (FTD) is a key component of the novel oral antitumor drug trifluridine/tipiracil, which is approved for the treatment of patients with metastatic colorectal cancer refractory to standard chemotherapies. A microRNA analysis of three colorectal cell lines was conducted to investigate causes of FTD resistance. Drug resistant sublines of DLD-1, HCT-116, and RKO cells were developed by continuous administration of increasing doses of FTD for 5 months. The let-7d-5p gene, which maps to chromosome 9q22.32, was downregulated in the FTD-resistant DLD-1 sublines. DLD-1 cells became more resistant to FTD when let-7d-5p was knocked down and more sensitive when let-7d-5p was overexpressed. The FTD-resistant sublines were not cross-resistant to 5-fluorouracil (5-FU); 5-FU sensitivity was affected only slightly when let-7d-5p as overexpressed or knocked down. These data indicate that let-7d-5p increases sensitivity of FTD but not 5-FU and that let-7d-5p is a potential clinical marker of treatment sensitivity.

## INTRODUCTION

Colorectal cancer (CRC) was the second leading cause of cancer death in both men and women in the 35 Organization for Economic Co-operation and Development countries in North and South America, Europe, and Asia-Pacific in 2015 [1]. The prognosis of metastatic (m)CRC is poor. Between 2004 and 2010 in the USA, the 5-year survival of patients with mCRC at diagnosis was only 13% [2]. Novel drugs and combination regimens for management of mCRC have increased median overall survival (OS) from 6 to over 30 months in the past two decades. 5-FU-based regimens have become the preferred mCRC treatment. Continuous infusion of 5-FU, leucovorin, and either irinotecan (FOLFIRI) or oxaliplatin (FOLFOX) have increased median OS to 20 months or more [3]. The addition of biologically targeted antibody drugs to infusion regimens has resulted in a median OS of up to 21 months [4], with it being 29 months in patients with additional lines of therapy [5]. Despite these recent advances, additional effective options would be especially beneficial for prolonging survival of patients who are refractory to current therapies.

Trifluridine/tipiracil is an oral combination therapy comprising trifluridine (FTD), which is a thymidine analog, and tipiracil, which is a thymidine phosphorylase inhibitor that improves the bioavailability of FTD [6]. FTD is incorporated directly into DNA, thereby interfering with its function [7]. The 5-FU metabolite fluorodeoxy-uridine (FdUrd) is incorporated into DNA and has an antiproliferative effect; however, the incorporation of FTD into DNA is reported be to significantly higher than that of 5-FU [8, 9]. An international, double-blind, placebo-controlled Phase III study confirmed the efficacy of trifluridine/tipiracil in patients with metastatic colorectal cancer (mCRC) who had previously been treated with standard chemotherapy [10]. FTD resistance has been reported *in vitro* in colorectal cell lines [11], and the involvement of micro (mi)RNAs in chemoresistance has been reported in several cancers [12, 13]. miRNAs are a conserved class of noncoding small RNAs that regulate the expression of genes involved in self renewal, survival, and tumor progression [14]. Several miRNAs that are aberrantly expressed in CRC have been associated with clinical outcomes [15].

In this study, we analyzed miRNA expression in CRC parental cell lines and FTD-resistant sublines to investigate the relationship of miRNAs and FTD treatment effectiveness. The miRNA let-7d-5p was associated with FTD resistance; both miRNA and mRNA expression differed in FTD-resistant and parental cells. miRNA and mRNA clustered in a locus on chromosome 9 (miRNA at 9p22.32) were downregulated in FTD-resistant cells, one of which was the miRNA let-7d-5p, a member of the let-7 family that is known to target oncogenes and several genes regulating the cell cycle and cell proliferation [16].

## RESULTS

### FTD-resistant colorectal cancer cell lines

**Figure 1:**
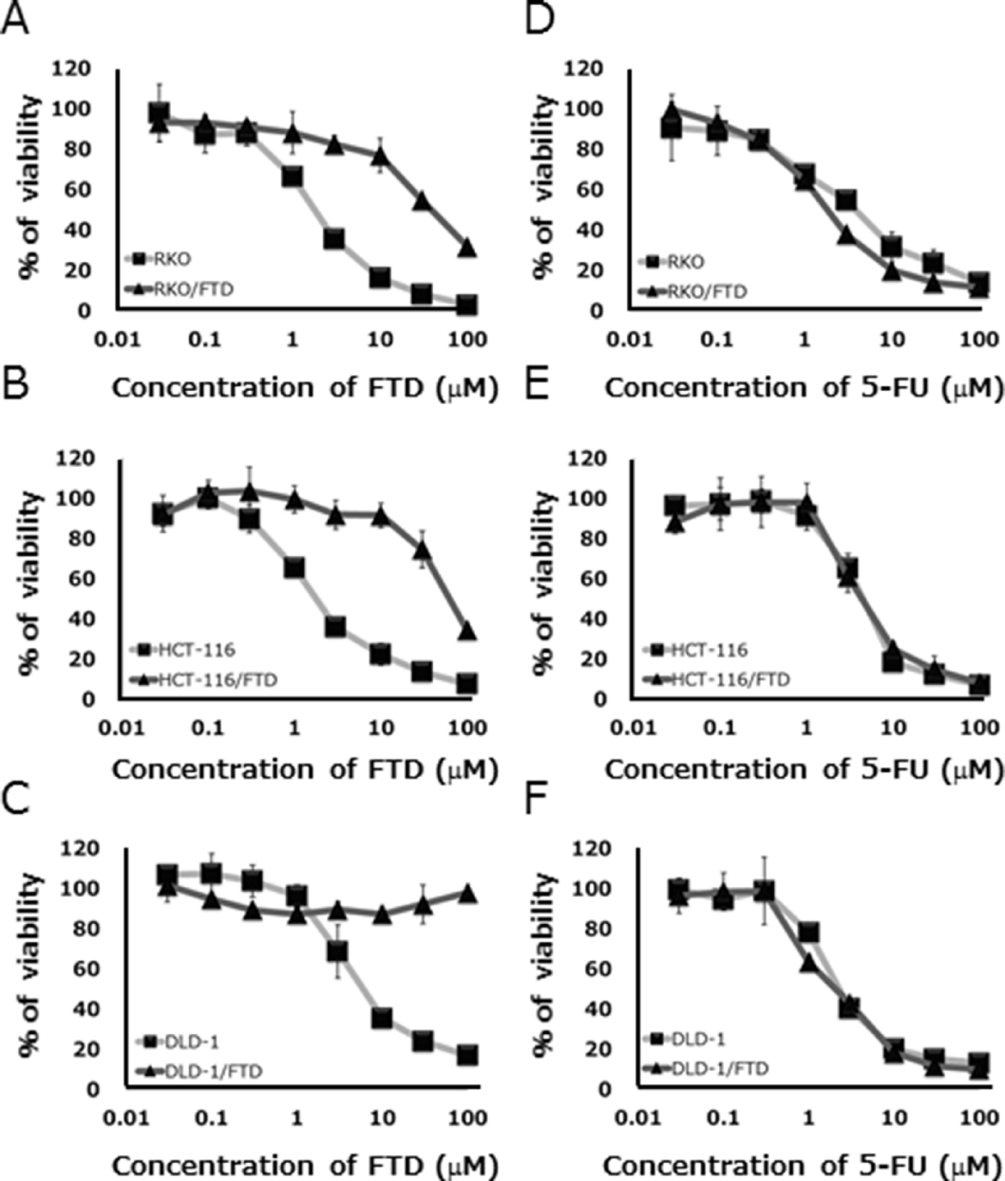
*In vitro* sensitivity of parental and trifluridine (FTD)-resistant cell lines to FTD and 5-FU Cell lines were cultured with various concentrations of FTD and 5-FU for 72 h. Data are means ± SD (*n* = 3). All FTD-resistant cell lines were less sensitive to FTD than the parent cell lines. (**A**) RKO, (**B**) HCT-116, (**C**) DLD-1, and were not cross-resistant to 5-FU. (**D**) RKO, (**E**) HCT-116, (**F**) DLD-1. Cell viability was assayed by crystal violet staining.

We established FTD-resistant sub lines of DLD-1 and HCT-116, and RKO CRC cells by exposure to step-wise increasing concentrations of FTD over 3–5 days starting at 1 μM and ending at 400 μM. FTD resistance was estimated as the ratio of the IC_50_ of each resistant line to that of the respective parent cell line after exposure to various concentrations of FTD for 3 days. Each of the cell lines had become highly resistant to FTD, with IC_50_ ratios that were 22.5- to > 40-fold higher than the IC_50_ of the parent cell lines (Figure 1A–1C, Supplementary Table 1). FTD-resistant sublines were not cross-resistant to 5-FU (Figure 1D–1F, Supplementary Table 1). These results suggest that different mechanisms of action were involved in development of FTD and 5-FU resistance.

### miRNA and mRNA expression in FTD-resistant cell lines

**Figure 2:**
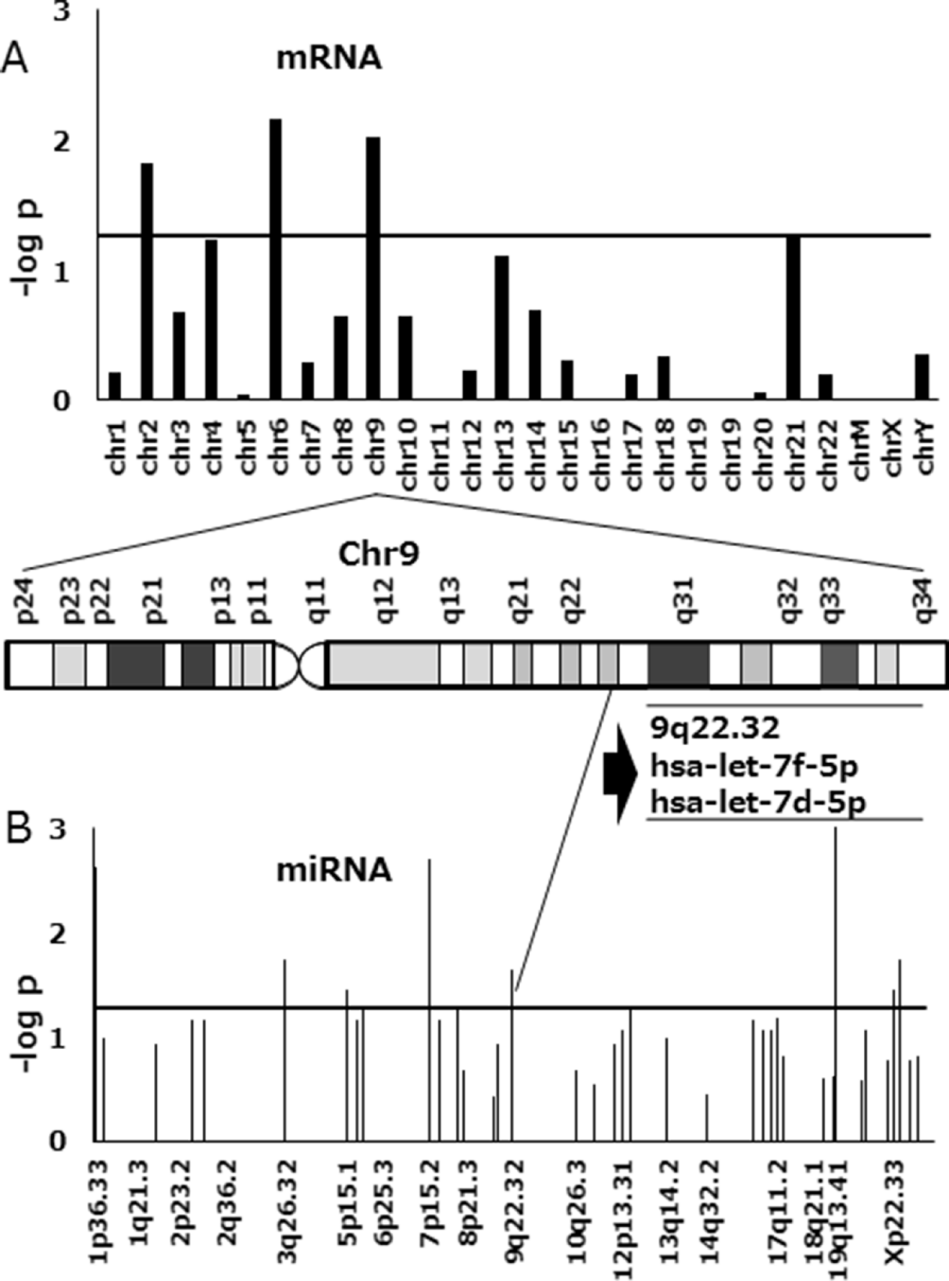
Manhattan plot of *p*-values in FTD-resistant DLD-1 colorectal cancer cells compared with the parental cell line (**A**) Association results are shown for mRNA and (**B**) miRNA. MiRNA and mRNA clustered in a genome locus on chromosome 9 (9p22.32) and were downregulated in FTD-resistant cell lines. One of the miRNAs was let-7d-5p. The horizontal axis includes chromosomal locations and the vertical axis includes the–log_10_ *p*-value of Fisher's exact probability test. The horizontal line indicates a *p*-value of 2 × 10^−3^.

To identify the miRNA associated with FTD resistance, we compared miRNA expression in resistant and sensitive cell lines using 3D-Gene human miRNA and mRNA oligo chips (Toray). Probe sets that downregulated with a fold-change greater than two were counted, and Fisher's exact probability test results with *p*-values of 2 × 10^−3^, were considered as chromosomal regions with a significant expression change. Of the three cell lines, DLD-1/FTD was the most resistant to FTD, and had acquired a significant downregulation of two miRNAs encoded by a gene located on 9q22.32 including let-7d-5p (Figure 2A). We also found significant downregulation of mRNA expressed by a gene located on chromosome 9 (Figure 2B). We therefore speculated that let-7d-5p was downregulated by a genome alteration that occurred during the acquisition of FTD resistance.

### let-7d expression in FTD-resistant and parental cell lines

**Figure 3:**
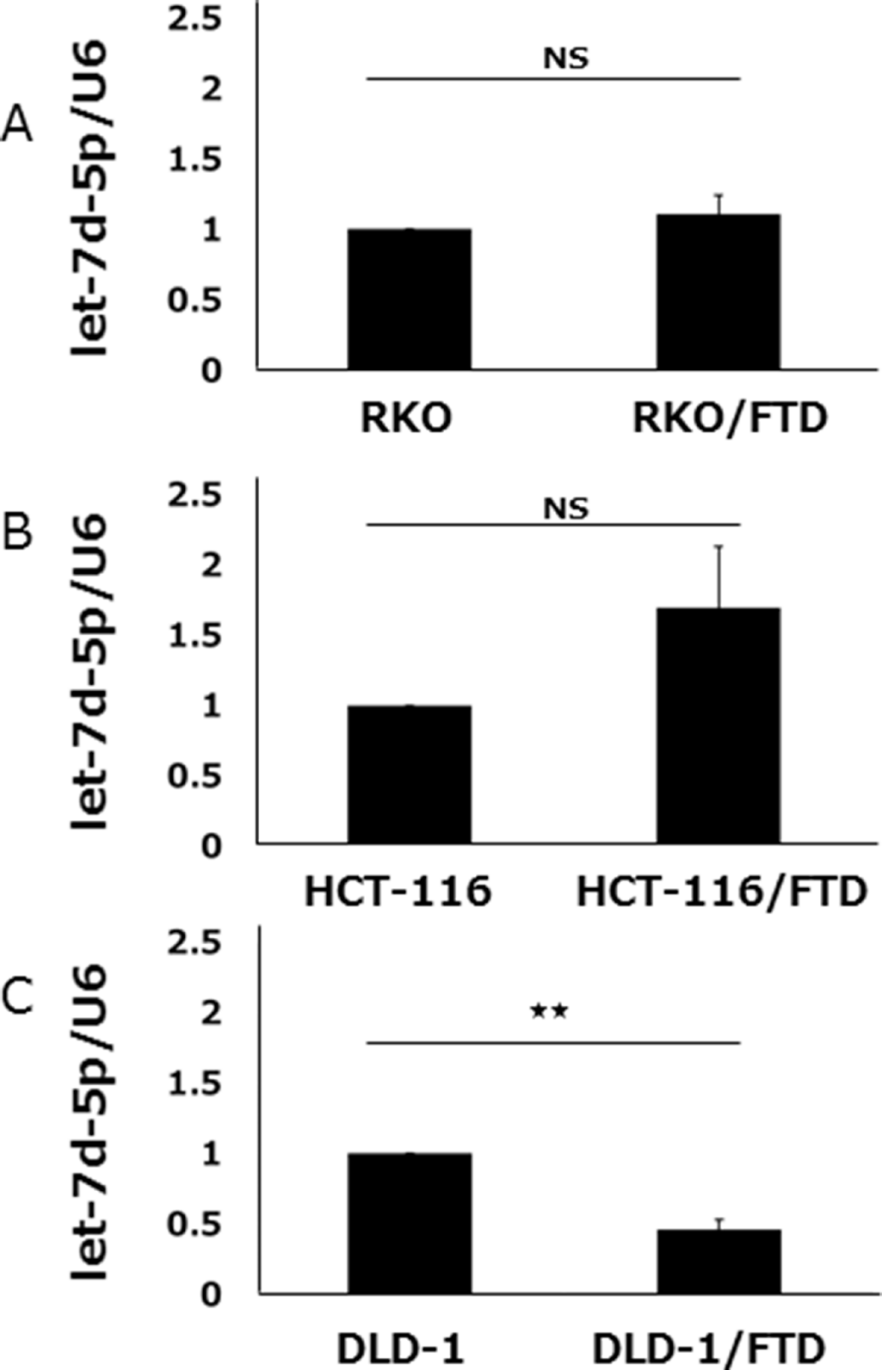
let-7d-5p expression of FTD-resistant and parental cell lines let-7d-5p levels in (**A**) RKO and RKO/FTD, (**B**) HCT-116, HCT-116/FTD, (**C**) DLD-1 and DLD-1/FTD cells were assayed by qRT-PCR and normalized against U6 snRNA (small nuclear ribonucleic acid). Data are means ± SE of duplicate determinations (**p < 0.01; NS, not significant).

qPCR was used to compare let-7d-5p expression in FTD-resistant and parental sublines. We found that let-7d-5p was downregulated in DLD-1/FTD compared with its parental cell line (Figure 3C), but that let-7d-5p expression was not significantly different in either HCT-116/FTD or RKO/FTD cells compared with the respective parental cells (Figure 3A, 3B).

### let-7d-5p controls sensitivity to FTD

**Figure 4:**
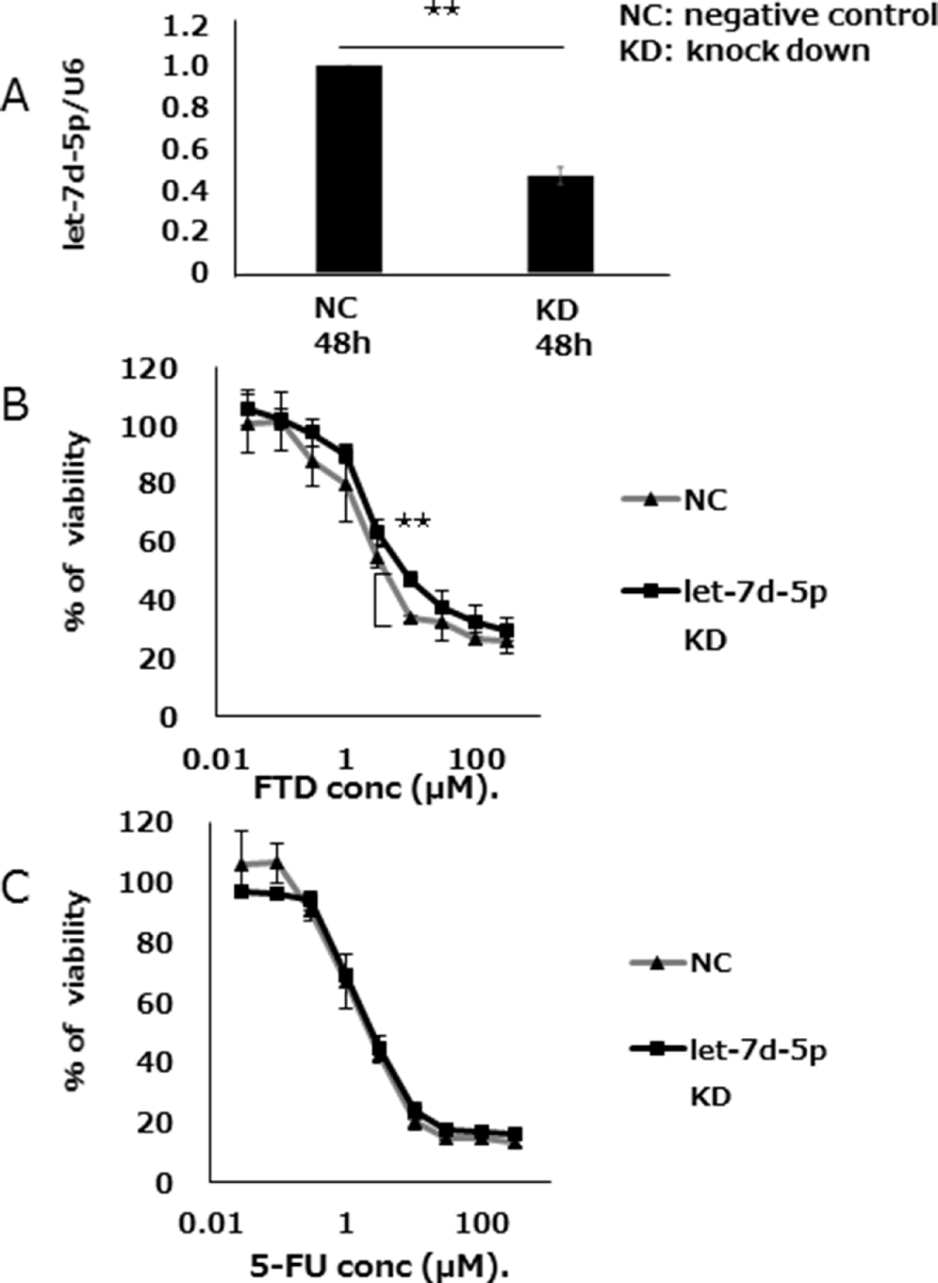
let-7d-5p knockdown inhibits FTD sensitivity (**A**) Expression of let-7d-5p was assayed by qPCR in both let-7d-5p inhibitor-transfected and untransfected cells. (**B**) Transfected cells were cultured with various concentrations of FTD and (**C**) 5-FU. Data are means ± SD (*n* = 3) (**p < 0.01). Cell viability was assayed by crystal violet staining.

We assessed the effects of let-7d-5p expression on FTD sensitivity by treating DLD-1 cells with anti-let-7d-5p oligo RNA. qPCR assay found that let-7d-5p expression was lower in cells treated with anti-let-7d-5p than those treated with an oligo RNA-negative control (Figure 4A). Anti-let-7d-5p-treated DLD-1 cells were less sensitive to FTD than DLD-1 negative control cells and had IC_50_ values of 16.8 μM and 7.6 μM, respectively (IC_50_ fold change = 2.2, Figure 4B and Supplementary Table 2). 5-FU sensitivity was slightly changed in anti-let-7d-5p-treated cells (Figure 4B, and Supplementary Table 2).

**Figure 5:**
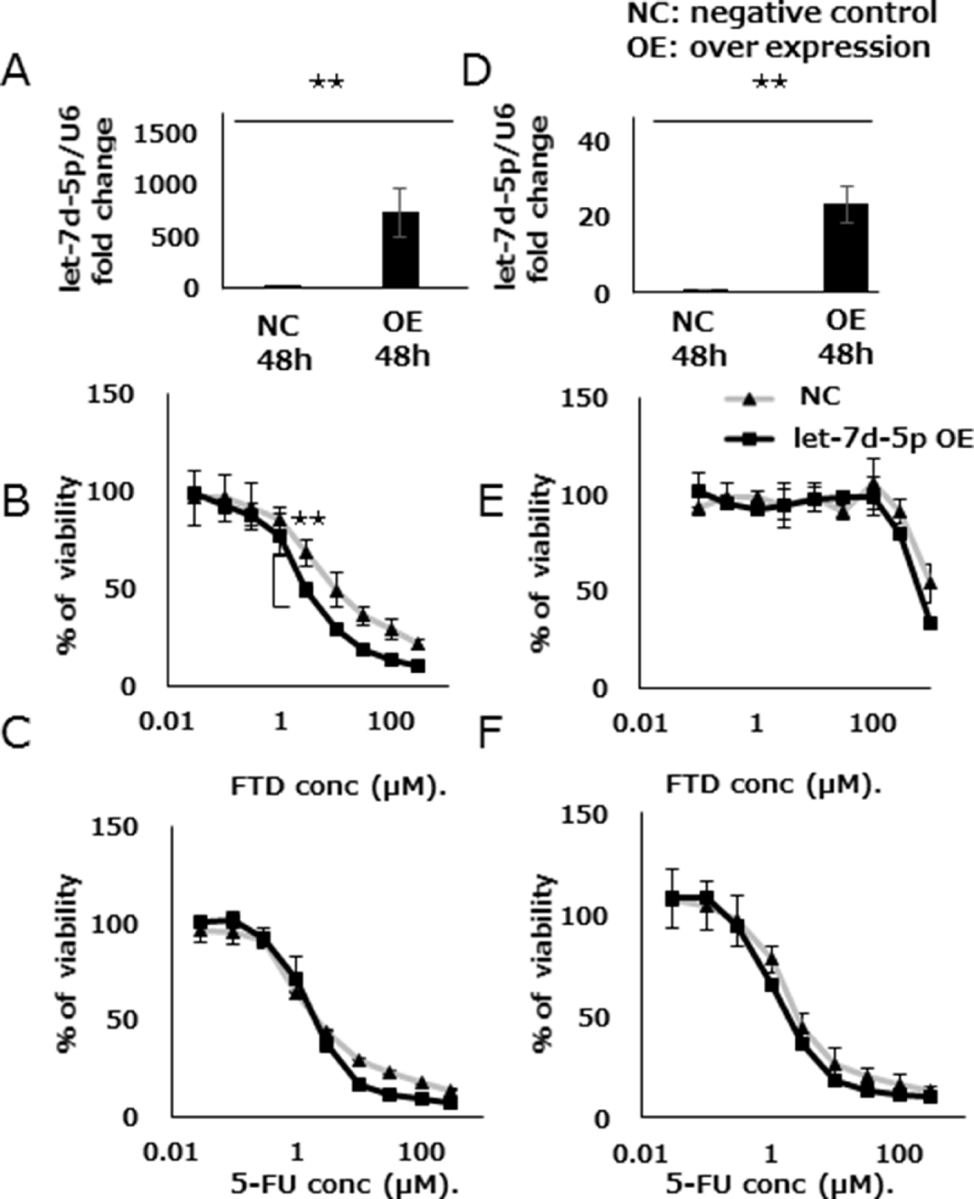
let-7d-5p overexpression enhances FTD sensitivity Expression of let-7d-5p was assayed by qPCR in both let-7d-5p mimic-transfected and untransfected cells (**A**) DLD-1, (**D**) DLD-1/FTD. Transfected cells were cultured with various concentrations of FTD in (**B**) DLD-1, (**E**) DLD-1/FTD, and 5-FU (C) DLD-1, (**F**) DLD-1/FTD. Data are means ± SD (*n* = 3) (**p < 0.01). Cell viability was assayed by crystal violet staining.

We assessed the effects of let-7d-5p overexpression on FTD sensitivity by treating DLD-1 with a let-7d-5p mimic. The expression of let-7d-5p was assayed by qPCR, which showed that let-7d-5p expression was higher in cells treated with the let-7d-5p mimic than in those treated with an oligo RNA-negative control (DLD-1 in Figure 5A; DLD-1/FTD in Figure 5D). Contrary to the results of treatment with the let-7d-5p inhibitor, DLD-1 cells treated with the let-7d-5p mimic which induced overexpression of let-7d-5p, were more sensitive to FTD than negative controls were. The IC_50_ values were 13.9 μM and 3.7 μM, respectively (IC_50_ fold change of 0.27), (Figure 5B and Supplementary Table 2). 5-FU sensitivity was slightly changed in cells treated with the let-7d-5p mimic compared with negative controls (Figure 5C and Supplementary Table 2). Both parental DLD-1 and FTD-resistant DLD-1/FTD cells were sensitized to FTD (Figure 5E and Supplementary Table 2) and slightly sensitized to 5-FU (Figure 5F and Supplementary Table 2) by the let-7d-5p mimic. These data suggest that let-7d-5p was more closely associated with FTD than with 5-FU sensitivity.

### Aurora B, a let-7d target, controls FTD sensitivity

**Figure 6:**
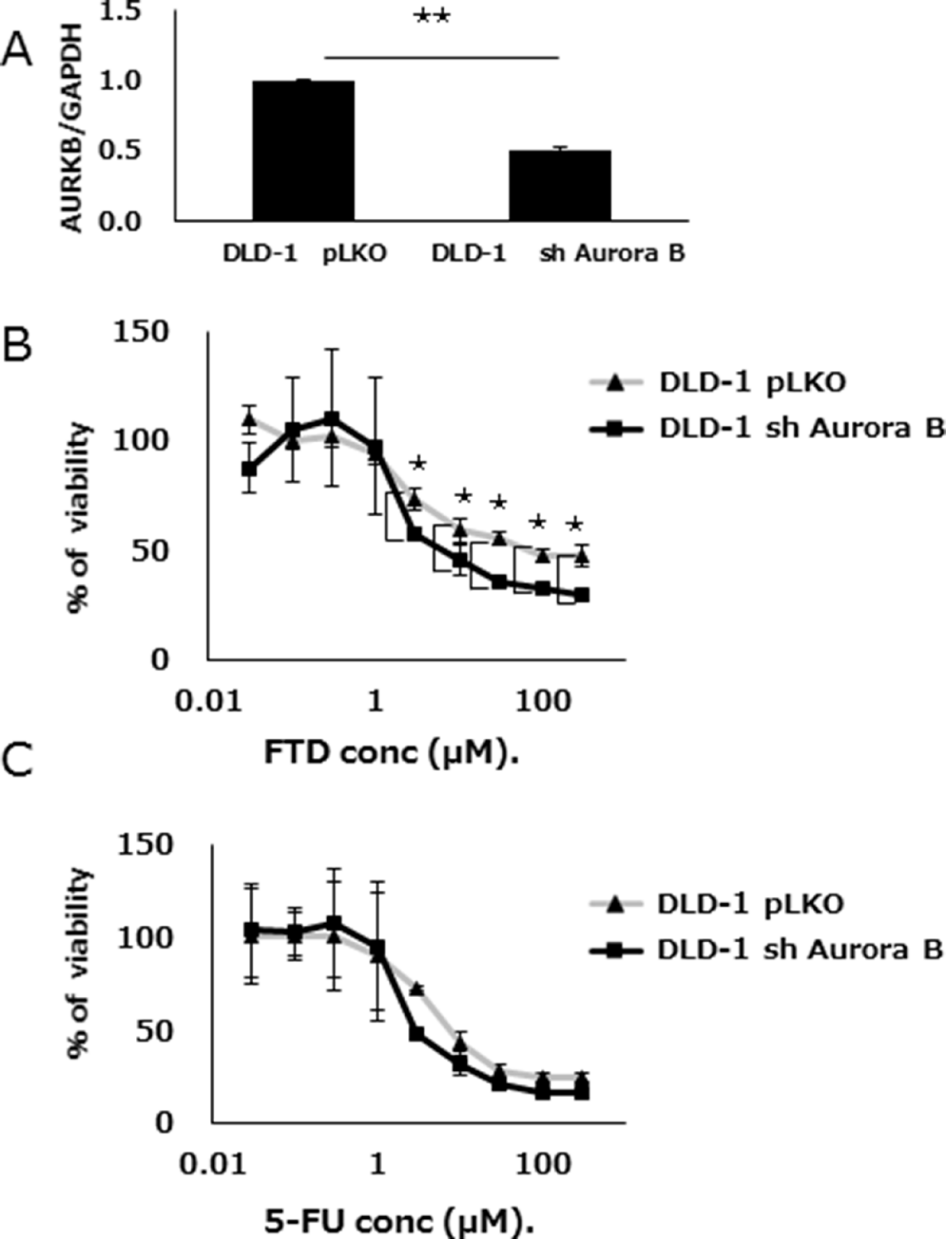
Aurora B knockdown enhances FTD sensitivity (**A**) Expression of Aurora B was assayed by qPCR in both shRNA knockdown Aurora B- or control vector (pLKO)-transfected DLD-1 cells. Data are means ± SE of replicate determinations (**p < 0.01). Transfected cells were cultured with various concentrations of (**B**) FTD, and (**C**) 5-FU. Data are means ± SD (*n* = 3) (*p < 0.05). Cell viability was assayed by crystal violet staining.

TargetScan target prediction software indicated that Aurora B was a possible let-7d-5p target (Supplementary Table 4). We found that let-7d-5p was downregulated and Aurora B upregulated, in DLD-1/FTD compared with DLD-1 cells (Supplementary Figure 1). Gene set enrichment analysis (GSEA) showed an RalA or RalB knockdown signature in DLD-1 versus DLD-1/FTD cells (Supplementary Figure 5). In those gene sets, upregulated genes included Aurora B and its substrate BIRC5 (Survivin), which are the components of a chromosomal passenger complex. Ral has been related to chromosomal passenger complex activity [17]. The chromosomal passenger complex Aurora B, INCENP, Borealin, and Survivin, which associate with chromosomes at prophase of mitosis and translocate to the spindle at anaphase/telophase were all upregulated in FTD-resistant cell lines (Supplementary Figure 4). We hypothesized that Aurora B, which is involved in cell division, is also involved in FTD resistance. DLD-1 cells were transfected with either a lentiviral Aurora B-specific shRNA or a control vector, treated with FTD or 5-FU, and cell viability was assayed. The knockdown efficacy of Aurora B was measured by qPCR. (Figure 6A). Aurora B knockdown cells were more sensitive than negative controls to FTD, with IC_50_ values of 15.7 μM and 82.7 μM, respectively (IC_50_ fold change = 0.19) (Figure 6B and Supplementary Table 3). 5-FU sensitivity was only slightly changed in Aurora B knockdown cells compared with negative controls (Figure 6C and Supplementary Table 3). Collectively, the data suggested that let-7d and Aurora B inhibition had an additive effect on FTD-induced antiproliferative activity.

## DISCUSSION

We showed that let-7d-5p was associated with FTD sensitivity. Let-7 is a well-known tumor suppressor, and its expression is downregulated in several cancers [16]. Restoration of endogenous let-7 expression has been found to prevent tumorigenesis [18]. FTD is a thymidine analog and is incorporated into DNA [8]. We predicted that genome alterations occur during acquired FTD resistance. Changes in expression of mRNA and miRNA located on chromosome 9 were the gene signature related to this biological phenotype, i.e., FTD resistance (Figure 2 and Supplementary Figure 6). We identified let-7d-5p in encoded by a gene located on 9q22.32, which is a fragile site locus and is occasionally deleted in colorectal cancer [19]. In our study, let-7d-5p could have been downregulated by genome deletion or loss of heterozygosity, but genome alteration analysis is needed for confirmation. To determine whether let-7d-5p was related to the FTD sensitivity, we assayed both cellular let-7d-5p expression and FTD sensitivity.

Let-7 miRNA regulates the expression of several oncogenes, including RAS and MYC [20, 21] to inhibit cancer cell proliferation and tumor development. Low-level let-7 expression occurs in many cell types, and the chromosomal region including human let-7 is deleted in many cancers [19]. In the NCI-60 colorectal cancer cell line, epithelial type cells have relatively high let-7d expression compared with embryonic mesenchymal cells [22].

Trifluridine/tipiracil was effective for the treatment of refractory metastatic colorectal cancer in clinical trials, but the mechanism of FTD- induced antiproliferative effects is not clearly understood. We showed that miRNA let-7d-5p was related to FTD-induced antiproliferative effects, and that its expression was inversely correlated with those effects. In contrast to FTD, a change in let-7d-5p expression had only a slight effect on 5-FU-induced antiproliferative effects. In preclinical studies, trifluridine/tipiracil demonstrated antitumor activity against both 5-FU-sensitive and resistant colorectal cancer cell lines [23]. This study showed that 5-FU had similar effects on FTD-resistant and parental cell lines. Let-7d-5p was thus more closely related to FTD than 5-FU sensitivity, and may reflect the mode of action of FTD-induced antiproliferative effects.

Trifluorothymidine-5′-triphosphate (F3dTTP) the active form of FTD is incorporated into DNA much more efficiently than FdUTP, the active form of 5-FU [8, 9]. FdUTP is also incorporated into RNA and contribute to cell damage, so the inhibition of RNA synthesis is involved in 5-FU rather than FTD sensitivity. Let-7d-5p may reflect DNA dysfunction, such as DNA damage-induced response, rather than inhibition of RNA synthesis. Consistent with this, let-7 miRNA are reported to target DNA replication pathways and DNA damage response-related apoptosis, but not RNA synthesis [16]. Let-7d-5p was downregulated in DLD-1 but not HCT-116 and RKO cells, indicating that the mechanism of acquired FTD resistance was cell type dependent and mediated by miRNA let-7d-5p. Some instances of FTD resistance originate in let-7d-5p downregulation, and let-7d-5p restoration can increase FTD sensitivity. In this study, only DLD-1 cells were sensitized by overexpression of let-7d-5p, but that might also occur in other cell types, such as HCT-116 and RKO. Even though origins of resistance may differ, the downstream pathways may overlap.

Let-7 family members share the same seed sequence, i.e., nucleotides 2–8 at the 5′ end, and often have similar functional consequences. This may explain why other family members, such as let-7a, let-7b, let-7c, let-7e, and let-7f, had expression patterns like those of let-7d in the DLD-1 cell microarrays (Supplementary Figure 2). We found two downregulated microRNAs (let-7d-5p and let-7f-5p) on the chromosome 9q22.32 region in FTD-resistant DLD-1 cells, but qPCR found that only let-7d-5p was significantly down regulated. Consequently, we investigated the function of let-7d-5p function in this study (Supplementary Figure 1).

Clinically, the study findings may have predictive and therapeutic value. First, low let-7d-5p expression was correlated with decreased trifluridine/tipiracil effectiveness. Therefore, assay of let-7d expression in tumor cells may assist in predicting drug effectiveness. Second, let-7d-5p overexpression was found to sensitize FTD-induced antiproliferative effects in both parental and FTD-resistant cell lines to a greater extent than 5-FU. Let-7d-5p expression profiling might be useful for allocating patients to genome information-based chemotherapy, and targeting let-7d with an appropriate delivery system might overcome FTD resistance. A recent report showed that reduced let-7d expression was associated with poor CRC outcomes [24], suggesting that let-7d-5p might be a useful in clinical practice as a biomarker. The key let-7d-5p targets in drug resistant tumor phenotypes have not been identified. In this study, let-7d-5p targets, such as HMGA2, that had high scores in the prediction software (TargetScan) were not significantly upregulated in resistant cell lines (Supplementary Figure 4). Other predicted let-7d-5p targets included *RAS* and *MYC*. *K*-*N*- and *HRAS* all have let-7d-5p binding sequences in their 3′-UTRs. Only *HRAS* was upregulated in DLD-1/FTD cells with downregulated let-7d-5p. *KRAS* was downregulated, and *NRAS* expression did not change. The data thus indicated that let-7d-5p-mediated *RAS* expression was compensated by another feedback loop. *KRAS* is involved in the sensitivity to anticancer agents in CRC, but FTD treatment has been reported to prolong survival independent of the presence of *KRAS* mutations [25], suggesting that *KRAS* was not involved in causing FTD resistance. In this study, *MYC* expression in DLD-1 and DLD-1/FTD cells was not different. *MYC* and let-7 have been reported to participate in a double negative feedback loop [26, 27]. If let-7d-5p-mediated *MYC* expression is not involved in FTD resistance, then other let-7d-5p targets need to be considered.

Aurora B has been considered as a molecular target of anticancer agents [28]. Its upregulation is followed by p53 phosphorylation and its degradation via the polyubiquitination–proteasome pathway and suppression of the expression of p53 target genes involved in cell cycle inhibition and apoptosis, including *Gadd45*, *PTEN*, *PUMA*, and *p21* [29]. These are known to be tumor suppressor genes, and were downregulated in the FTD-resistant cell lines in our study (Supplementary Figure 4).

The results indicated that FTD resistance was partially related to Aurora B. Let-7b binds to the Aurora B kinase 3′ UTR and inhibits expression of the kinase mRNA and protein [30]. Much like let-7b, nucleotides 2–8 at the let-7d 5′ UTR match the 3′ UTR sequence of the Aurora B kinase (Supplementary Figure 3). Let-7b overexpression induced mitotic defects characteristic of those caused by Aurora B interference, including increased rates of polyploidy, multipolarity, and premature spindle assembly checkpoint inactivation, leading to forced exit from chemically induced mitotic arrest [30]. Let-7d-5p induced mitotic defects, or p53 activation characteristic of Aurora B perturbation, might be antiproliferative effects induced by FTD. The use of let-7d-5p as a predictive marker and its additive effect with trifluridine/tipiracil warrant further investigation.

## MATERIAL AND METHODS

### Parental and FTD-resistant cell lines

The RKO, HCT-116, and DLD-1 human colon cancer cell lines were obtained from the American Type Culture Collection (Manassas, VA, USA) and grown in Dulbecco's modified Eagle's medium (Sigma-Aldrich, MO, USA) supplemented with 10% fetal bovine serum (Thermo Fisher Scientific, MA, USA), 100 U mL^−1^ penicillin, and 100 UmL^−1^ streptomycin (Life Technologies, CA, USA). Cells were grown at 37°C in a humidified atmosphere with 5% CO_2_. FTD-resistant cells (RKO/FTD, HCT-116/FTD, and DLD-1/FTD) were established from each parent cell line by repeated, continuous (3- to 5-day) exposure of the cell cultures to escalating concentrations of FTD for about 5 months. Resistant cell lines were maintained as above. Cells were checked for short tandem repeats before use, and all experimental procedures were performed with exponentially growing cells.

### Chemicals

FTD and 5-FU were purchased from Tokyo Chemical Industry (Tokyo, Japan).

### Cytotoxicity assay

Cell lines were seeded at a density of 2,000 cells per well into 96-well plates and precultured for 24 h. They were then exposed to various concentrations of FTD and 5-FU for 72 h. The *in vitro* antiproliferative effects of FTD and 5-FU were evaluated by crystal violet staining. Cells were fixed with 4% glutaraldehyde for 20 minutes, stained with 0.05% crystal violet in 20% methanol for 20 minutes, and then rinsed in tap water. The plates were dried on paper for 1 hour, and 100 μL of a 1:1v/v mixture of ethanol and 0.1 mol L^−1^ sodium phosphate was added to each well. Absorbance was measured at 595 nm by an EnSpire multimode plate reader (PerkinElmer). The FTD and 5-FU concentrations that inhibited cell growth by 50% (IC_50_) were calculated from the regression lines. FTD and 5-FU resistance was estimated by dividing the FTD or 5-FU IC_50_ of the FTD-resistant cell line by the FTD or 5-FU IC_50_ of the corresponding parental cell line.

### miRNA and mRNA microarray analysis

Total RNA was isolated from cells of line with an miRNeasy mini kit (Qiagen), following the manufacturer's instructions. Microarray analysis of miRNA and mRNA expression were performed by 3D-Gene microarray (Toray). The raw data are available on the Gene Expression Omnibus Website (http://www.ncbi.nlm.nih.gov/geo/) using the SuperSeries accession number GSE96787 and includes both the mRNA (GSE96785) and microRNA expression profiles (GSE96786).

### Transfection

The miRNA mimics of let-7d (hsa-let-7d, 5′-AGAGGUAGUAGGUUGCAUAGUU-3′), and miRNA negative control (5′-UCACAACCUCCUAGAAAGA GUAGA-3′) were obtained from GeneDesign, Inc. (Osaka, Japan). The MiRNA inhibitor of let-7d (mirVana™ miRNA Inhibitors), and miRNA inhibitor negative control were obtained from Applied Biosystems. (Thermo Fisher Scientific K.K., USA). DLD-1 cells were transfected with 10 nM of miRNA mimics or 30 nM of miRNA inhibitors using Lipofectamine 3000 (Invitrogen, CA, USA) following the manufacturer's protocol.

### Short hairpin (sh)RNA construct and lentivirus production

The shRNA construct was provided by Lewis Cantley (Harvard Medical School) and cloned into the lentivirus vector pLKO. The oligonucleotide sequence of the shRNA targeting *AURKB* was 5′-CCGG- CCTGCGTCTCTACAACTATTT-CTCGAG-AAATAGT TGTAGAGACGCAGG-TTTTT-3′. The vector was cotransfected into 293T cells along with expression vectors containing the *gag/pol*, *rev*, and *vsvg* genes. Lentivirus was harvested 48 h after transfection, and 5 μg mL^−1^ polybrene was added. DLD-1 cells were infected with harvested lentivirus and selected with 2 μg mL^−1^ puromycin for 1 week.

### Real-time quantitative reverse-transcription polymerase chain reaction (qPCR)

To assay miRNA and mRNA expression, cDNA was first synthesized from extracted total RNA, and qPCR was then performed using a Mir-X miRNA First-Strand Synthesis and SYBR qRT-PCR Kit (Clontech). Relative expression was calculated by the CT-based calibrated standard curve method. The calculated values were then normalized against the expression of U6 small nuclear (sn)RNA for miRNA and GAPDH for mRNA.

### Statistical analysis

Statistically significant differences were determined by Student's *t*-test and Fisher's exact probability test as appropriate. Statistical analysis was performed using JMP Pro 9 (SAS Institute, Cary, NC, USA). Fisher's exact probability test with a *p*-value of 2 × 10^−3^ was used to select chromosomal regions with significant differential miRNA and mRNA expression. Probe sets that were downregulated in DLD-1 cells with a fold change of < 50% were counted and analyzed for statistical significance.

## SUPPLEMENTARY MATERIALS FIGURES AND TABLES


