# Supplementary material for: MicroRNA profiles involved in trifluridine resistance
Source: Oncotarget. 2017 May 23;8(32):53017–27. doi: 10.18632/oncotarget.18078 (PMC5581089; doi:10.18632/oncotarget.18078)
Supplement: Supplementary file 1 [file oncotarget-08-53017-s001.pdf]

MicroRNA profiles involved in trifluridine resistance

Supplementary Materials

Methods for FTD resistant cell line establishment

FTD-resistant cell lines (DLD-1/FTD, HCT-116/FTD, and RKO/FTD) were established from each parent cell line by repeated, continuous (3- to 5-day) exposure of the cell cultures to escalating concentrations of FTD for about 5 months, starting with 1 µM and final concentration is 400 µM. Cell lines were maintained in DMEM (Sigma-Aldrich, MO, USA) with 10% fetal bovine serum (FBS; Thermo Fisher Scientific, MA, USA). All cell lines were incubated at 37°C in a humidified atmosphere of 95% air and 5% CO_2.

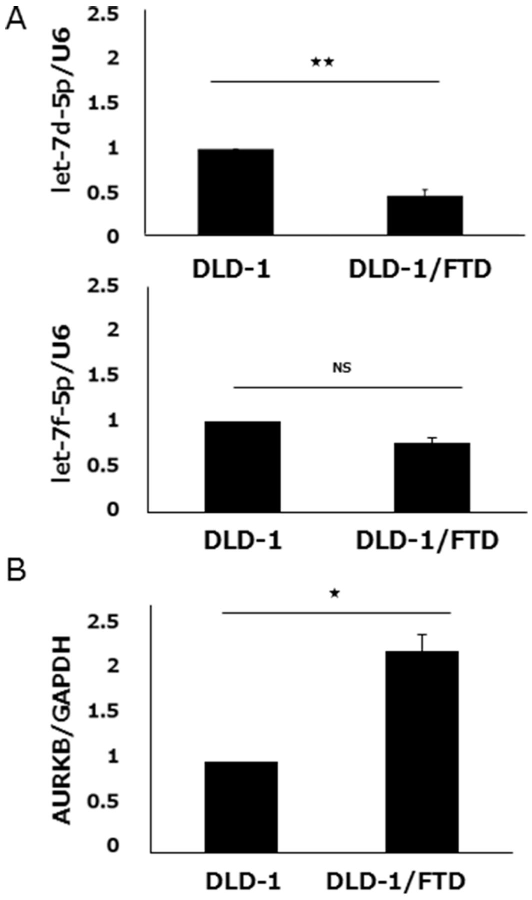

Supplementary Figure 1: let-7d-5p and Aurora B expression by FTD-resistant and parental cell lines. (**A**) let-7d-5p and let-7f-5p levels and (**B**) Aurora B in DLD-1 and DLD-1/FTD cells were assayed by qPCR and normalized against U6 snRNA expression in let-7d-5p, let-7f-5p and GAPDH in Aurora B. Data are means ± SE of at least one duplicate determination (*$p < 0.05$; **$p < 0.01$).

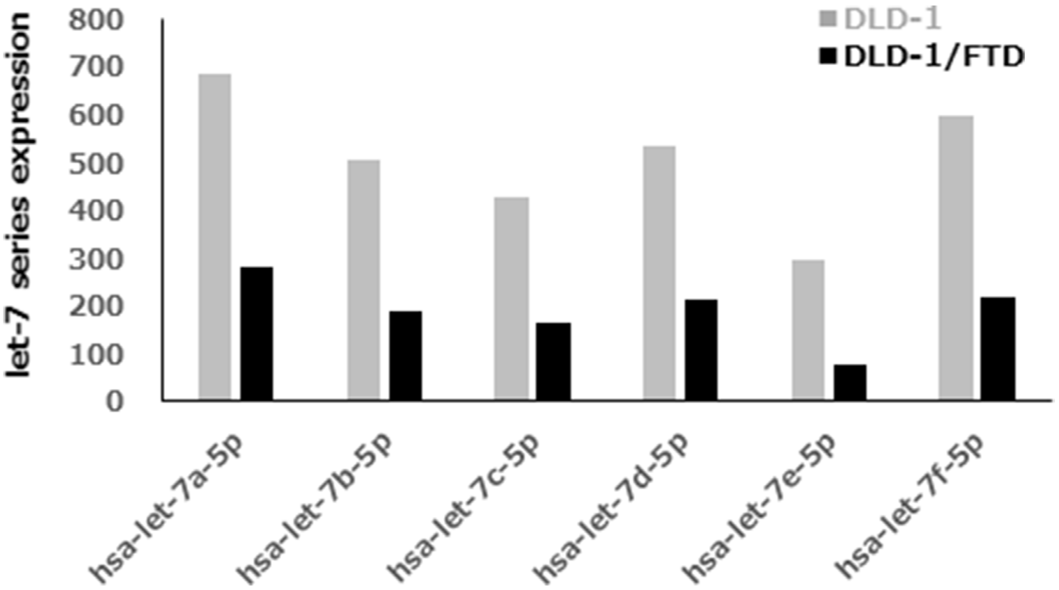

Supplementary Figure 2: Expression of let-7d-5p and other let-7 family members in FTD-resistant and parental cell lines. let-7 family expression in DLD-1 and DLD-1/FTD cells was assayed in microarrays. Microarray data were normalized against the median of the entire signal.

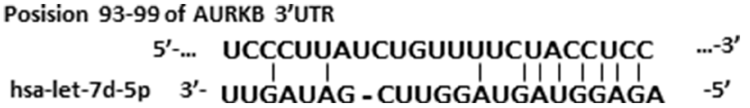

Supplementary Figure 3: predicted let-7d-5p 3′-UTR binding site to of Aurora B mRNA. The predicted binding site of let-7d-5p on Aurora B (AURKB) is shown; the binding sequence of let-7d-5p is underlined.

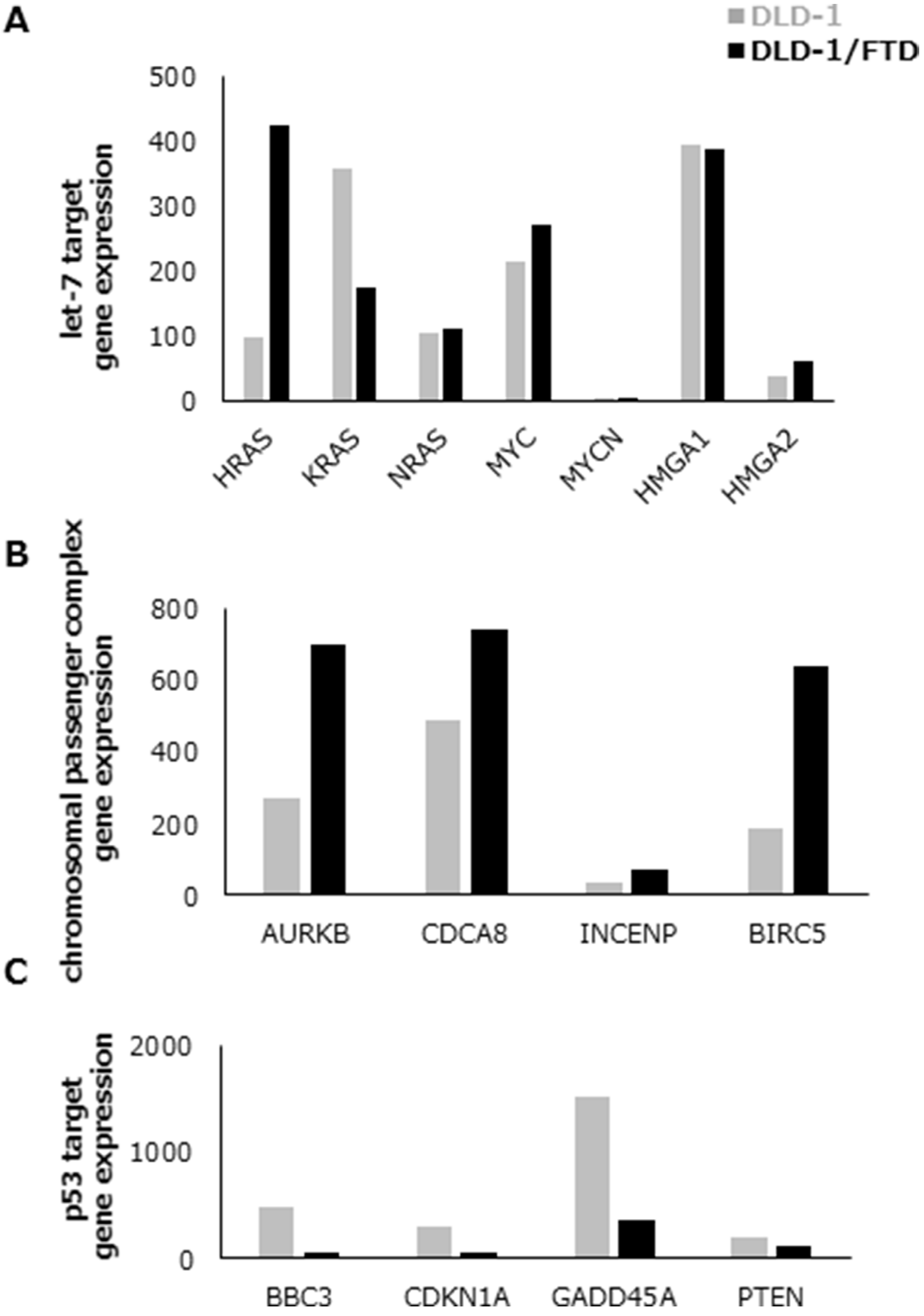

Supplementary Figure 4: mRNA expression profile of DLD-1 and FTD-resistant DLD-1 cells. mRNA expression in DLD-1 and DLD-1/FTD cells in microarrays. (**A**) Predicted targets of let-7d-5p genes and (**B**) chromosomal passenger complex genes, and (**C**) p53 target genes involved in cell cycle inhibition and apoptosis are shown. Microarray data were normalized against the median of the entire signal.

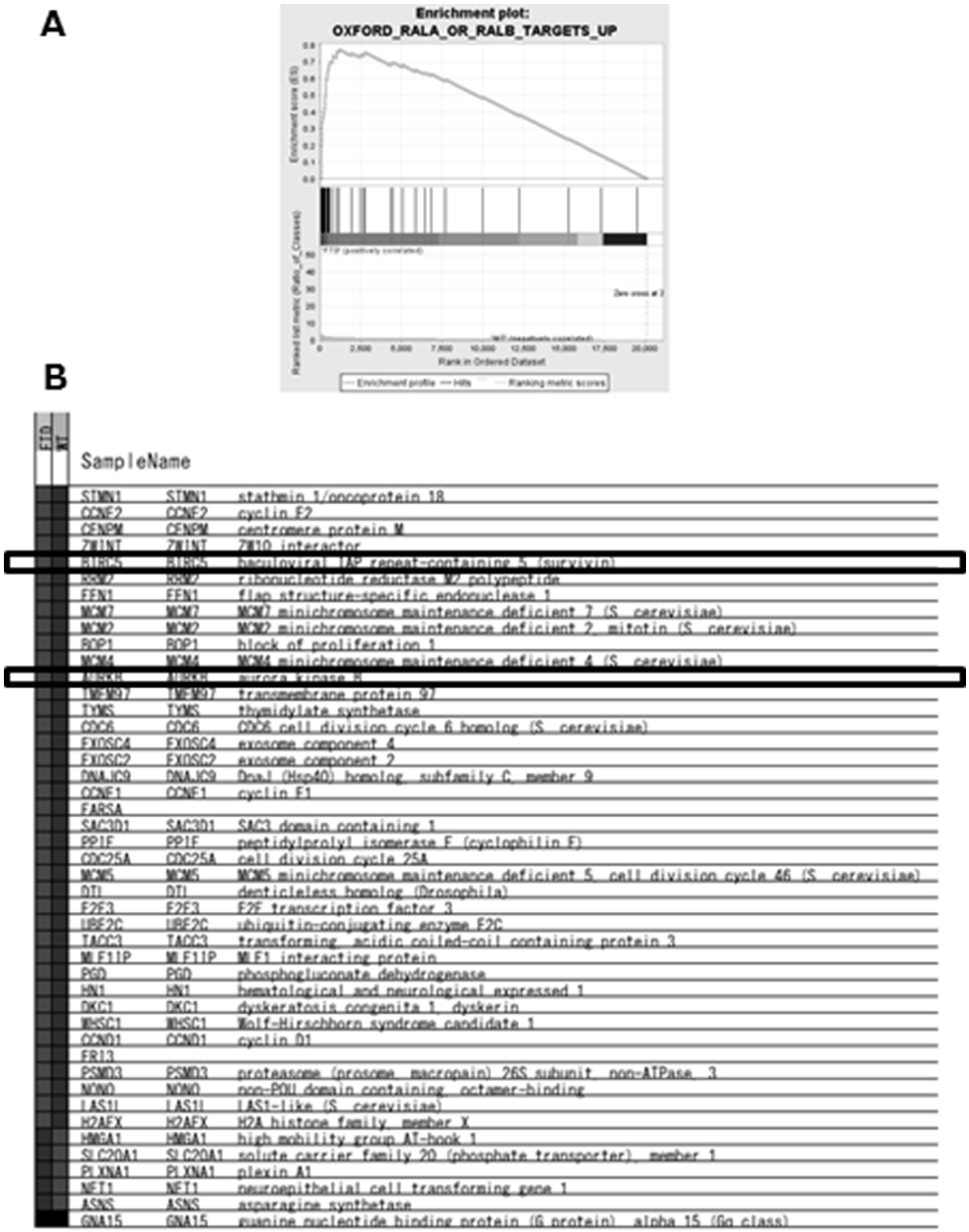

Supplementary Figure 5: Gene set enrichment analysis (GSEA) in DLD-1 and FTD-resistant DLD-1. (**A**) GSEA result for the RalA or RalB knockdown signature in DLD-1 versus DLD-1/FTD. (**B**) Upregulated gene sets in (A) include Aurora B and Survivin.

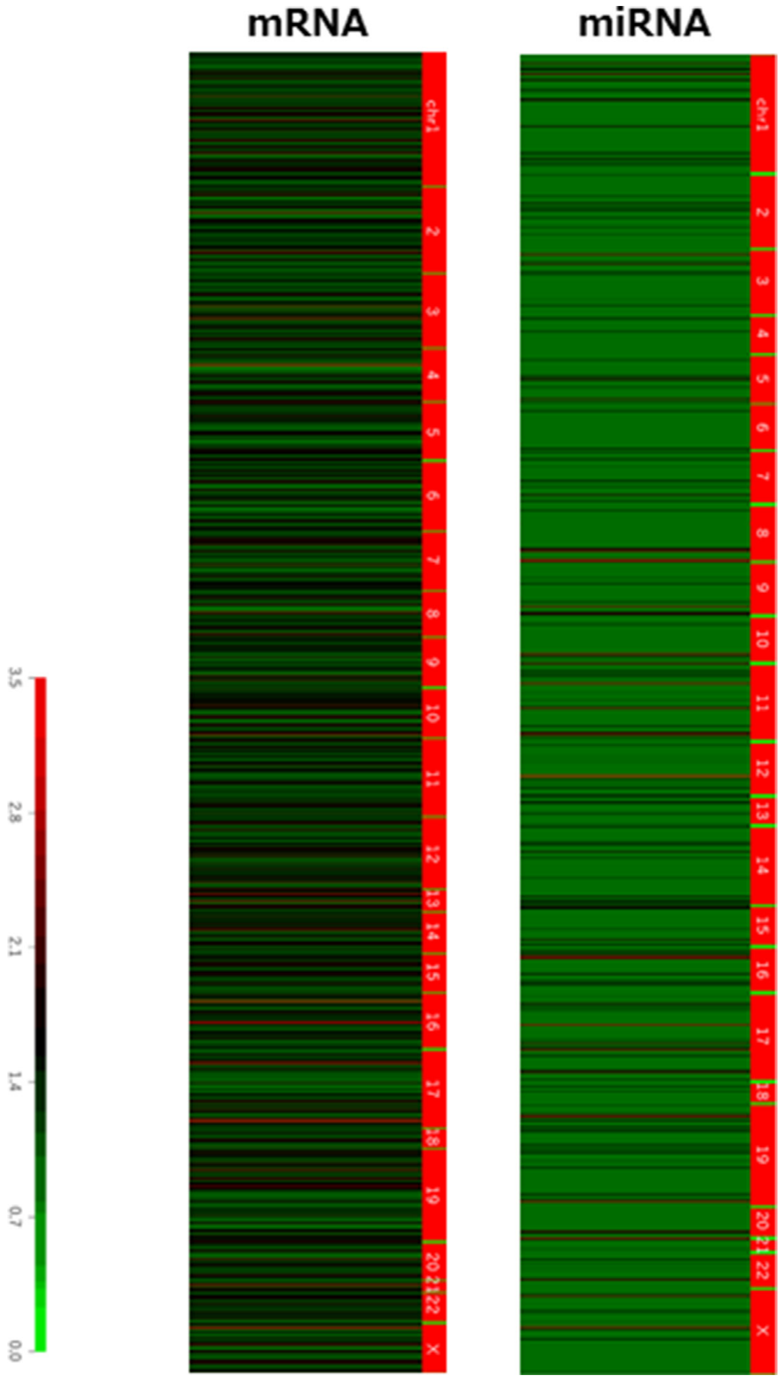

Supplementary Figure 6: Heatmap of gene expression change in genome view of DLD-1 and FTD-resistant DLD-1. Heatmap of mRNA (left) and miRNA (right) expression ratio between DLD-1 and FTD-resistant DLD-1 in the genome view. The value of the ratio exceeding 3.5 was set to 3.5.

Supplementary Table 1: Sensitivity of parent and trifluridine (FTD)-resistant cell lines to FTD and 5-FU

Cell line	IC_{50} of FTD (μM)		Degree of resistance to FTD	IC_{50} of 5-FU (μM)		Degree of resistance to 5-FU
	Parent cell lines	FTD-resistant cell lines		Parent cell lines	FTD-resistant cell lines	
RKO	1.8	40.6	22.5	3.7	2	0.5
HCT-116	2.1	65.4	36.3	4.4	4.8	1.1
DLD-1	7.5	> 300	> 40	2.8	2.3	0.8

IC_{50} values of FTD and 5-FU 72 h after treatment (i.e., the FTD concentration in μM that inhibit cell growth by 50%).

Supplementary Table 2: Sensitivity of let-7d-5p inhibitor-treated and let-7d-5p mimic-treated cell lines to FTD and 5-FU

cell line	Let-7d-5p treatment	IC_{50} of FTD (μM)		Degree of resistance to FTD	IC_{50} of 5-FU (μM)		Degree of resistance to 5-FU
		Negative control	Let-7d-5p inhibitor or mimic		Negative control	Let-7d-5p inhibitor or mimic	
DLD-1	inhibitor	7.6	16.8	2.2	2.5	2.9	1.2
DLD-1	Mimic	13.9	3.7	0.27	3.1	2.2	0.7
DLD-1/FTD	Mimic	> 1000	661.7	< 0.7	3.5	2.1	0.6

IC_{50} values of FTD and 5-FU 72 h after treatment (i.e., the FTD concentration in μM that inhibit cell growth by 50%).

Supplementary Table 3: Sensitivity of shRNA knockdown Aurora B and control vector-transfected cell lines to FTD and 5-FU

transfected vector	IC_{50} of FTD (μM)		Degree of resistance to FTD	IC_{50} of 5-FU (μM)		Degree of resistance to 5-FU
	Negative control	sh Aurora B		Negative control	sh Aurora B	
DLD-1	82.7	15.7	0.19	10.1	5.0	0.5

IC_{50} values of FTD and 5-FU 72 h after treatment (i.e., the FTD concentration in μM that inhibit cell growth by 50%).

Supplementary Table 4: Results of bioinformatic research about microRNAs targeting Aurora-B

miRNA	Position in the UTR	seed match	context++ score
hsa-let-7a-5p	563-569	7mer-m8	−0.39
hsa-let-7c-5p	563-569	7mer-m8	−0.39
hsa-let-7f-5p	563-569	7mer-m8	−0.39
hsa-let-7e-5p	563-569	7mer-m8	−0.39
hsa-let-7g-5p	563-569	7mer-m8	−0.39
hsa-let-7b-5p	563-569	7mer-m8	−0.38
**hsa-let-7d-5p**	**563-569**	**7mer-m8**	**−0.38**
